# Relationship between the triglyceride-glucose index and the SYNTAX score 2 in patients with non-ST elevation myocardial infarction

**DOI:** 10.1097/XCE.0000000000000277

**Published:** 2023-01-05

**Authors:** Onur Baydar, Alparslan Kilic, Erol Gursoy

**Affiliations:** Department of Cardiology, Koc University Hospital, Istanbul, Turkey

**Keywords:** NSTEMI, SYNTAX score 2, triglyceride-glucose index

## Abstract

We evaluated if admissiontriglyceride-glucose index (TyG index) correlated with the anatomical synergy between percutaneous coronary intervention with taxus and cardiac surgery (SYNTAX) score 2 in non-ST elevation myocardial infarction (NSTEMI), nondiabetic patients.

**Methods:**

SYNTAX score 2 (SSII) was retrospectively evaluated in 260 nondiabetic patients hospitalized with NSTEMI who underwent coronary angiography. The TyG index was calculated using the following equation: log [fasting triglycerides (mg/dl) × fasting glucose (mg/dl)/2]. We stratified patients according to tertiles of SSII (≤21.5, 21.5–30.6, and ≥30.6). These score ranges were defined as SSII low, SSII mid, and SSII high, respectively.

**Results:**

The average age of the patients was 57.2 ± 10.9 years; 135 patients (52.2%) were males. The average TyG index was 8.68 ± 0.12, and SSII was 18.9 ± 9.9. A moderate correlation was found between TyG index and SSII (*r* = 0.347; *P* < 0.001) and TyG index was independent risk factors for SSII high [odds ratio (OR), 6.0; 95% CI, 2.7–17.0; *P* < 0.001].

**Conclusion:**

In nondiabetic patients with NSTEMI, TyG index correlated with the SSII.

## Introduction

Coronary artery diseases (CAD) are the most common leading cause of death whole world [1]. Insulin resistance (IR) is one of the most important risk factors for CAD. IR decreases sensitivity to the metabolic effect of insulin and is associated with an increased risk of dyslipidemia, hypertension (HT), and hyperglycemia [2]. These metabolic abnormalities are strongly associated with acceleration of atherosclerosis by causing inflammation, vasoconstriction, and thrombosis [3].

Recent studies have demonstrated that the triglyceride-glucose (TyG) index is associated with IR, such as identified by homeostasis model assessment of IR (HOMA-IR) and hyperinsulinemic-euglycemic clamp testing. So, the TyG index is a simple and dependable biomarker of IR [4].

IR has a prognostic significance in patients with CAD, especially among those with non-ST elevation myocardial infarction (NSTEMI). It is not clear that IR is correlated with CAD severity.

The anatomical synergy between percutaneous coronary intervention (PCI) with taxus and cardiac surgery (SYNTAX) Score 1 (SSI) was created as part of the SYNTAX trial [5,6] in order to objectively characterize CAD, stratifying patients into low-, intermediate-, and high-risk groups [7]. However, the SSI has limitations like the lack of clinical variables, a personalized approach to decision-making, and predictive ability in the coronary artery bypass grafting subset of patients. The SYNTAX Score 2 (SSII) overcomes these limitations, by incorporating prognostically clinical variables and producing an individualized estimate of mortality risk associated with each revascularization strategy [8]. Subsequently, age, sex, creatinine clearance (CrCl), left ventricular ejection fraction (LVEF), peripheral artery disease, and chronic obstructive pulmonary disease were added for the calculation of the SSII [8–10].

In our study, we observed the TyG index correlation with the SSII in nondiabetic patients. The aim of the study was to evaluate the TyG index is useful for predicting advanced CAD as measured by the SSII.

## Methods

We prospectively observed 350 consecutive patients with NSTEMI undergoing PCI at the Avicenna Hospital Cardiology Department between January 2015 and January 2018. NSTEMI was defined according to the current guidelines [11]. We excluded patients with severe valvular heart disease, severe or decompensated heart failure, intra-aortic balloon pressure support requirement, severe renal failure, undergoing urgent cardiac surgery for revascularization, with known diabetes mellitus (DM), and treated with any DM medication. The study was approved by the Local Ethics Committee. In all patients, the TyG index was calculated using the following formula, logarithm of fasting TG × fasting glucose/2 [12]. Patients were divided into two groups according to their TyG index. The first group had patients with a TyG index of less than 8.65 and the second group had patients with a mean TyG index of at least 8.65. The cutoff value was based on the International Diabetes Federation definition [13]. HT was defined as blood pressure of more than 140/90 mmHg or being on treatment with antihypertensive medications. DM was defined as fasting glucose levels of more than 126 mg/dl or treatment with oral antidiabetic drugs or insulin. Hyperlipidemia was defined by reference to current guidelines [14]. Serum creatinine concentration was observed at hospital admission, every day for the following days, and at hospital discharge. Estimated glomerular filtration rate was calculated using the modified formula of Levey *et al.* [15]. Coronary angiography was performed by standard techniques. The catheterization films were examined for severity of CAD according to the SSII [8,9]. The anatomical-based SSI was determined (pre-PCI) for every coronary angiogram taken at study entry, by a trained analyst blinded for patient characteristics and outcome using the SS calculator (www.syntaxscore.org). The calculation of the anatomical-based SS has been described previously [6]. Subsequently, data on the baseline variables age, sex, CrCl, LVEF, peripheral artery disease, and chronic obstructive pulmonary disease were collected for the calculation of the SSII. We used the original SSII Calculator (www.syntaxscore.org) to obtain all SSII values. The algorithm of the SSII calculation has been described in detail elsewhere [8,9]. For our analysis, we stratified patients according to tertiles of SSII [8] (≤21.5, 21.5–30.6, and ≥30.6). The score ranges are referred to as SSII low, SSII mid, and SSII high, respectively. The Global Registry of Acute Coronary Events (GRACE) score was calculated using the GRACE risk calculator available on the website (https://www.outcomes-umassmed.org/grace/acs_risk2/index.html).

### Statistical analysis

All analyses were performed using SPSS version 26 for Windows (SPSS Inc, Chicago, Illinois, USA). Categorical data were compared using the Chi-square or Fisher’s exact test. Continuous variables are presented as mean ± SD. All variables were subjected to Kolmogorov–Smirnov testing to determine whether they were normally distributed. The independent samples *t*-test was used to compare the values of continuous variables between the two groups. One-way ANOVAtest was used for the analysis of more than two numeric variables. Correlation between different variables was assessed by Pearson’s correlation test for parametric variables and Spearman’s test for nonparametric variables. To evaluate the different factors affecting the severity of CAD (SSII) and to evaluate the effects of various factors on mortality development, we performed regression analyses using the Logistic Regression method. Variables with an unadjusted two-sided *P* < 0.05 were considered significant.

## Results

We retrospectively analyzed 260 consecutive NSTEMI patients referred for coronary angiography. None of our patients had a medical history of DM, and all had an HbA_1c_ of less than 6.0%. The average age of the patients was 57.2 ± 10.9 years; 135 patients (52.2%) were males. The average of fasting glucose was 115.1 ± 52.8 mg/dl and HbA_1c_ was 5.8 ± 0.9%. SSII was 18.9 ± 9.9, and GRACE score was 123.7 ± 10.8. Tables 1 and 2 describe the cardiac risk factors, medications, and laboratory blood results. The inhospital clinical course of patients is detailed in Table 3. We stratified patients according to tertiles of SSII [8] (≤21.5, 21.5–30.6, and ≥30.6). The score ranges are referred to as SSII low (192; 73.1%), SSII mid (33; 12.3%), and SSII high (35; 14.6%), respectively. There was NS difference between the three groups in terms of age, weight, and sex. HT, TyG index, and fasting glucose levels were only detected significantly higher in the SSII high group (Table 4).

**Table 1:** Main characteristics of the patients

Variables	Values
Age (years)	57.2 ± 10.9
Men, *n* (%)	135 (52.2)
Weight (kg)	75.9 ± 5.7
HT, *n* (%)	130 (61.0)
HL, *n* (%)	94 (37)
CVA, *n* (%)	18 (7.1)
Smoker, *n* (%)	73 (28.1)
Previous Myocardial Infarction, *n* (%)	80 (32.0)
Creatinine (mg/dl)	1.0 ± 0.9
EF, *n* (%)	53.7 ± 7.7
eGFR (ml/min/1.73 m²)	92.0 ± 16.0
Troponin peak (ng/dl)	2.2 ± 0.9
Fasting Glucose (mg/dl)	115.1 ± 52.8
HbA_1c_, (%)	5.8 ± 0.9

CVA, cerebrovascular accident; EF, ejection fraction; eGFR, estimated glomerular filtration rate; HbA_1c_, glycosylated hemoglobin; HL, hyperlipidemia; HT, hypertension.

**Table 2:** Medications taken before catheterization

Medications	Values
Aspirin, *n* (%)	120 (46.6)
Clopidogrel, *n* (%)	21 (8)
Statins, *n* (%)	86 (33.6)
ACE-I/ARB, *n* (%)	135 (51.8)
Beta-blockers, *n* (%)	93 (36.6)
CCB, *n* (%)	26 (10.5)
Nitrates, *n* (%)	44 (16.5)

ACE-I, angiotensin converting enzyme inhibitor; ARB, angiotensin receptor blocker; CCB, calcium channel blocker.

**Table 3:** Inhospital clinical course of patients

Variables	Values
Time to reperfusion (h)	4.8 ± 2.2
Length of hospital stay (days)	4.8 ± 1.5
Inhospital mortality, *n* (%)	10 (4.0)
Treated with PCI, *n* (%)	187 (72.7)
Treated with CABG, *n* (%)	26 (10.7)
Medical treatment, *n* (%)	31 (12.4)

CABG, coronary artery bypass grafting; PCI, percutaneous coronary intervention.

**Table 4:** Baseline clinical and procedural characteristics of patients according to SSII groups

Variables	SSII low (*n* = 192)	SSII mid (*n* = 33)	SSII high (*n* = 35)	*P*
Age (years)	57.3 ± 10.7	56.3 ± 12.0	57.0 ± 11.3	0.84
Men, *n* (%)	95 (50)	18 (55.8)	21 (60.8)	0.32
Weight (kg)	75.6 ± 5.9	75.4 ± 5.8	77.3 ± 4.6	0.13
HT, *n* (%)	119 (63.7)	14 (41.9)	22 (64.7)	0.02
HL, *n* (%)	73 (37.1)	10 (30.2)	14.3 (41.2)	0.54
CVA, *n* (%)	12 (6.3)	5 (14)	2(5.9)	0.17
Smoker, *n* (%)	58 (30.1)	7 (20.9)	8 (25.5)	0.41
Previous myocardial infarction, *n* (%)	61 (32.4)	12 (34.9)	8 (25.5)	0.55
TyG index	8.6 ± 0.1	8.6 ± 0.1	8.8 ± 0.1	<0.001
Fasting glucose (mg/dl)	108.0 ± 46.2	106.6 ± 45.9	158.0 ± 67.6	<0.001
LDL (mg/dl)	124.1 ± 35.9	136.7 ± 53.1	126.2 ± 45.3	0.17
GRACE score	123.2 ± 10.6	125.4 ± 12.1	124.2 ± 10.7	0.44
HDL (mg/dl)	43.7 ± 9.8	45.0 ± 10.5	42.1 ± 11.0	0.38
EF, *n* (%)	53.9 ± 7.6	53.5 ± 8.2	53.5 ± 7.6	0.91
eGFR (ml/min/1.73 m²)	88.6 ± 16.9	88.3 ± 15.5	92.0 ± 16.0	0.39

CVA, cerebrovascular accident; EF, ejection fraction; eGFR, estimated glomerular filtration rate; GRACE, The Global Registry of Acute Coronary Events; HL, hyperlipidemia; HT, hypertension; PCI, percutaneous coronary intervention; SSII, SYNTAX score 2; TyG index, triglyceride-glucose index.

A moderate correlation was found between TyG index and SSII (*r* = 0.347; *P* < 0.001), and TyG index was independent risk factor for SSII high [odds ratio (OR), 6.0 95% CI, 2.7–17.0; *P* < 0.001] (Table 5).

**Table 5:** Independent risk factors of SSII high in logistic regression analysis

Variables	OR (95% CI)	*P*
TyG index	6 (2.7–17.0)	<0.001
Weight (kg)	1.0 (0.9–1.2)	0.052
Grace score	0.9 (0.9–1.1)	0.62
HT	1.3 (0.5–2.4)	0.87
HL	1.2 (0.6–2.6)	0.63

CI, confidence interval; HL, hyperlipidemia; HT, hypertension; OR, odds ratio; TyG index, triglyceride-glucose index.

## Discussion

In our study, a significant relationship between the SSII and the TyG index was demonstrated. To the best of our knowledge, this is the first report of the association between the TyG index and SSII in nondiabetic patients.

Previous studies show that IR is a cardiovascular risk factor in patients with and without DM [16]. Hyperglycemia is a strong independent predictor for short-term mortality among patients with the acute myocardial infarction with no relation to DM status [17,18]. Procoagulant and inflammatory effects of IR may also cause accelerated atherosclerosis [19,20]. IR can be described as the HOMA-IR and TyG index [21,22]. CAD progression is a chronic disorder; therefore, IR as a specific marker of chronic dysglycemia and is more strongly related to chronic CAD than fasting glucose levels. The mechanism of vascular damage by hyperglycemia includes endothelial dysfunction, oxidative stress and overproduction of reactive oxygen species high levels of free fatty acids and oxidized LDL cholesterol, increase in cytokine activation and inflammatory response, increased sympathetic activity and blood pressure, and increased platelet activation and increased risk for thrombotic events [23–29]. Some studies have also shown that the TyG index could be an independent predictor for subclinical atherosclerosis in the general population [30–33]. In another study, Lee *et al*. [3] have shown that a higher TyG index is related to a raised risk of coronary artery stenosis in asymptomatic patients with type 2 diabetes, especially when they have risk factors for CAD. Thus, in our study, we evaluated the TyG index to determine correlation with SSII in nondiabetic NSTEMI patients.

The SSI has been extensively studied for a variety of clinical outcomes in different patient populations [34,35] including patients with NSTEMI [36,37] or STEMI [38]. The more recently developed SSII has been complemented with clinically significant prognostic variables such as age, sex, CrCl, LVEF, peripheral artery disease, and chronic obstructive pulmonary disease, known to be independent predictors of mortality at 4 years in patients with stable CAD enrolled in the SYNTAX trial [39,40]. In the SYNTAX trial patient population, this was translated into better discrimination of risk for long-term mortality for SSII compared with the SSI [8,9]. Therefore, SSII is more useful to show individualized estimate of mortality risk associated with each revascularization strategy. Additionally, a recent study showed that SSII demonstrated to be an independent predictor for 4.5-year all-cause mortality in patients with one or two vessel diseases [41]. In our study, we showed that TyG index correlated with CAD severity and was independent risk factors for the SSII high group. In light of this data, we can suggest that the TyG index may have the potential to be used in different populations as a marker of IR and can give information about CAD.

Our study had some limitations. First, it was a single-center study. Second, the study cohort was relatively small. Finally, the study has the deficiency of follow-up data.

In conclusion, in nondiabetic patients with NSTEMI, TyG index correlated and were associated with the SSII. Future follow-up studies are needed to verify our results and to provide useful cutoffs to define patients at increased risk.

## Acknowledgements

All the authors contributed to: (a) substantial contributions to conception and design, or acquisition of data, or analysis and interpretation of data, (b) drafting the article or revising it critically for important intellectual content, and (c) final approval of the version to be published.

### Conflicts of interest

There are no conflicts of interest.
